# Implementation of perioperative FLOT compared to ECX/EOX chemotherapy regimens in resectable esophagogastric adenocarcinomas: an analysis of real-world data

**DOI:** 10.2340/1651-226X.2024.35431

**Published:** 2024-05-14

**Authors:** Kristian Egebjerg, Tobias Sørup Andersen, Lene Bæksgaard, Rajendra Garbyal, Mette Siemsen, Michael Achiam, Morten Mau-Sørensen

**Affiliations:** a Department of Oncology, Copenhagen University Hospital – Rigshospitalet, Copenhagen, Denmark; b Department of Pathology, Copenhagen University Hospital – Rigshospitalet, Copenhagen, Denmark; c Department of Thoracic Surgery, Copenhagen University Hospital – Rigshospitalet, Copenhagen, Denmark; d Department of Surgery and Transplantation, Copenhagen University Hospital – Rigshospitalet, Copenhagen, Denmark; e Department of Clinical Medicine, University of Copenhagen, Copenhagen, Denmark

**Keywords:** Gastric cancer, esophageal cancer, adenocarcinoma, FLOT, EOX, perioperative chemotherapy, Real-World Evidence

## Abstract

**Background and purpose:**

Perioperative 5-FU, leucovorin, oxaliplatin, and docetaxel (FLOT) is recommended in resectable esophagogastric adenocarcinoma based on randomised trials. However, the effectiveness of FLOT in routine clinical practice remains unknown as randomised trials are subject to selection bias limiting their generalisability. The aim of this study was to evaluate the implementation of FLOT in real-world patients.

**Methods:**

Retrospectively collected data were analysed in consecutive patients treated before or after the implementation of FLOT. The primary endpoint was complete pathological response (pCR) and secondary endpoints were margin-free resection (R0), overall survival (OS), relapse-free survival (RFS) tolerability of chemotherapy and surgical complications.

**Results:**

Mean follow-up time for patients treated with FLOT (*n* = 205) was 37.7 versus 47.0 months for epirubicin, cis- or oxaliplatin, and capecitabine (ECX/EOX, *n* = 186). Surgical resection was performed in 88.0% versus 92.0%; pCR were observed in 3.8% versus 2.4%; and R0 resections were achieved in 78.0% versus 86.0% (*p* = 0.03) in the ECX/EOX and FLOT cohorts, respectively. Survival analysis indicated no significant difference in RFS (*p* = 0.17) or OS (*p* = 0.37) between the cohorts with a trend towards increased OS in performance status 0 (hazard ratio [HR] = 0.73, 95% confidence interval [CI]: 0.50–1.04). More patients treated with ECX/EOX completed chemotherapy (39% vs. 28%, *p* = 0.02). Febrile neutropenia was more common in the FLOT cohort (3.8% vs. 11%, *p* = 0.0086). 90-days mortality (1.2% vs. 0%) and frequency of anastomotic leakage (8% vs. 6%) were equal and low.

**Interpretation:**

Patients receiving FLOT did not demonstrate improved pCR, RFS or OS. However, R0 rate was improved and patients in good PS trended towards improved OS.

## Introduction

Esophageal, gastroesophageal junction (GEJ), and gastric adenocarcinomas (ADC), collectively known as esophagogastric ADC, are among the most common malignancies worldwide.

The incidence of esophageal and GEJ ADC is on the rise [1, 2]. The treatment of esophagogastric ADC depends on the disease stage. Patients with localised disease are potentially curable when surgically resected. In contrast, patients with metastatic disease are managed with systemic therapy, including chemotherapy alone or in combination with trastuzumab or immune checkpoint inhibitors, depending on the expression of biomarkers [3, 4].

Various approaches for curative-intended treatment of locally advanced esophagogastric ADC exist worldwide. Management strategies include neoadjuvant chemoradiotherapy as well as perioperative chemotherapy followed by surgical resection [5]. The benefit of perioperative chemotherapy was established two decades ago [6]. The pivotal MAGIC trial demonstrated an improvement from 23 to 36% in 5-year survival with perioperative ECF (Epirubicin, Cisplatin, and Fluorouracil [5-FU]) compared to surgery alone [7]. Efforts to achieve further improvement succeeded with the practice-changing presentation of the FLOT4-AIO trial at the annual American Society of Clinical Oncology (ASCO) meeting in 2017 [8]. The introduction of docetaxel as part of the FLOT regimen (5-FU, leucovorin, oxaliplatin, and docetaxel) improved 5-year survival by an additional 9%, increasing from 36 to 45%, compared to ECX/ECF (Epirubicin, Cisplatin, and Capecitabine/5-FU) [9]. Consequently, FLOT was promptly implemented at our institution on the 21st of November 2017, replacing the previously used ECX/EOX (Epirubicin, Cisplatin/Oxaliplatin, Capecitabine).

Evidence-based guidelines are established on data derived from well-designed randomised trials. However, the narrow eligibility criteria in these trials may lead to selection bias, thereby limiting the generalisability of the data obtained from randomised clinical trials (RCTs) [10]. The aim of this study was to evaluate whether the superiority of the FLOT regimen compared to ECX/EOX translated into clinical benefits in a consecutive cohort of patients representing real-world data. The two regimens were compared in terms of complete pathological regression, margin-free resection, overall survival (OS), relapse-free survival (RFS), tolerability of chemotherapy, and surgical complications

## Methods

### Design

This was a retrospective study of a cohort of consecutively treated patients at a single institution using a prespecified analytic plan. In February 2020, the plan for the collection of clinical variables and statistical analyses, including the selection of endpoints, was completed. Subsequently, the database was established, and data were captured by review of medical charts.

### Endpoints

The primary endpoint evaluating efficacy was complete pathological regression (pCR) defined as no residual tumour cells in the excised primary tumour or lymph nodes (ypT0N0). In cases where tumour cells were absent in the surgical specimen, a complete histopathological work-up of the entire GEJ was conducted. This included an additional 15 mm length from both ends as part of standardised procedures. For poorly cohesive carcinoma, immunohistochemistry using cytokeratin staining (MOC-31/Ep-CAM) was performed to identify residual tumour cells.

Secondary endpoints evaluating efficacy included margin-free resection defined as no microscopic cancer cells at the tumour resection margin (R0 resection); postsurgical pathological T and N (ypTN) stages; tumour shrinkage on computed tomography (CT) scans prior to surgery; OS defined as the time from cancer diagnosis to death; RFS defined as time from cancer diagnosis to relapse or death.

Secondary endpoints evaluating the tolerability of chemotherapy encompassed hospitalisation due to neutropenic fever, the use of Granulocyte Colony Stimulating Factor (G-CSF). Additionally, they included discontinuation of chemotherapy, dose reductions, start of postoperative chemotherapy, and completion of pre- and postoperative cycles of chemotherapy.

Secondary endpoints evaluating surgical complications included the Clavien-Dindo score, anastomotic leakage, and days admitted postoperatively

### Sample size

A sample size sufficient to identify a difference in pCR of 10% (16% vs. 6%), based on the results of the FLOT4-AIO trial [11], with a power of 80% and a one-sided *p*-value of 0.05 (type 1 error rate) was calculated. Considering a dropout rate of 15%, a total sample size of 280 patients, with 140 patients in the ECX/EOX group and 140 patients in the FLOT group, would be needed.

### Treatment procedure

Patients treated with the ECX/EOX regime were treated with a modified version of the chemotherapy schedule used in the REAL-2 trial [12]. This consisted of three cycles before surgery and three cycles after surgery, administered every 3 weeks. The dosages of each cycle were 50 mg/m^2^ epirubicin (E) intravenously, 500 mg/m^2^ oral capecitabine (X) twice daily in 14 days and either 60 mg/m^2^ cisplatin (C) or 130 mg/m^2^ oxaliplatin (O), both administered intravenously. The FLOT cohort received four cycles of preoperative and four cycles of postoperative 50 mg/m^2^ docetaxel (T), 85 mg/m^2^ oxaliplatin (O), 200 mg/m^2^ leucovorin (L), and 2,600 mg/m^2^ 5-FU (F), all given intravenously every 2 weeks as described in the FLOT4-AIO trial [9]. Chemotherapy was postponed, reduced, or discontinued if unacceptable toxicity occurred. Other reasons for altering chemotherapy included patient preference and disease progression. G-CSF was administered almost exclusively to patients in the FLOT group following guidelines for the administration of the FLOT regimen. A CT scan was performed after the completion of preoperative chemotherapy in both groups to assess tumour changes and confirm resectability. Surgery was planned 4 weeks after the last cycle of preoperative chemotherapy. Patients with esophageal and GEJ cancer underwent transthoracic esophagectomy with or without interposition. Patients with gastric cancer underwent total or subtotal gastrectomy.

### Study population

Patients were identified from the Danish EPIC-Electronic Healthcare Medical Records by searching for those who underwent curative ECX/EOX or FLOT treatment at the Department of Oncology, Rigshospitalet. The implementation of FLOT took place on November 21, 2017. Patients in the ECX/EOX cohort were included from May 2013 to November 2017, and those receiving FLOT from November 2017 to June 2020. In total, the identification process yielded more patients than initially estimated to be necessary according to the sample size calculation. Consequently, we decided to include all identified patients to enhance statistical power.

### Data collection

Data were collected from medical records and manually extracted by health-educated personnel trained according to standard operating procedures for data capture using the Research Electronic Data Capture (REDCap) software (https://redcap.regionh.dk/). Tumour location was determined based on diagnostic gastroscopy. Pathology reports were reviewed by specialists in esophagogastric cancer (LB, MMS, RG, MA) to validate the pCR rate. Reasons for dose modifications or discontinuation of chemotherapy were assessed using a checklist of predefined expected adverse events, allowing for the selection of more than one reason for chemotherapy adjustments. Follow-up time was defined as the duration from the diagnostic biopsy until death or the last recorded date the patient was registered as alive. Follow-up was updated as of the 29th of November 2023.

### Data analysis

Chi-square test was employed to compare categorical variables, while unpaired t-test was used to compared means for numerical variables. The exact Poisson method was utilised to calculate two-sided 95% confidence intervals (CI). Kaplan-Meier survival analysis was conducted to assess overall and RFS. For further analysis of potential variations in OS benefit within specific subgroups, a subgroup analysis was performed. Additionally, a multivariate Cox proportional hazards regression model was utilised to evaluate the HR based on baseline characteristics. This approach allowed for the assessment of differences in survival between cohorts when corrected for these characteristics, as well as an examination of the effect size of each factor. RStudio (version 1.4.1717) was used for data analysis.

### Ethical approval

The Danish Patient Safety Authority (Case no.: *31-1521-202)* approved the collection of data from patient journals by the 26^th^ of March 2020.

## Results

### Cohort

**Figure 1:**
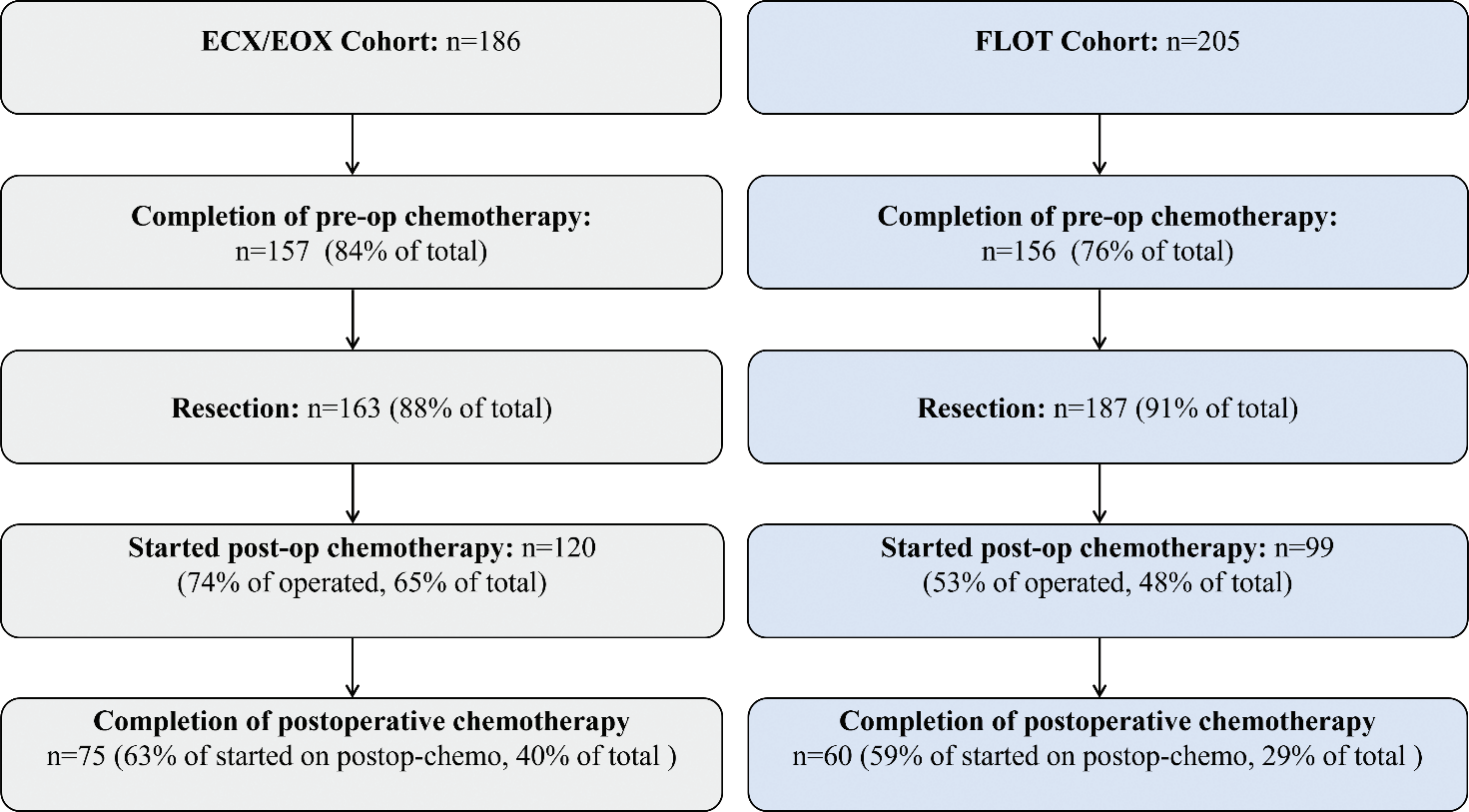
Summary of the treatment/patient flow in the ECX/EOX and FLOT cohorts. *‘Completion’ is defined as administration of all scheduled cycles of chemotherapy regardless of dose reduction.

A total of 391 patients were included, with 186 and 205 patients in the ECX/EOX and FLOT cohorts, respectively. The patient flow is summarised in Figure 1. One patient switched from FLOT to ECX after 1 cycle of FLOT due to neuropathy, and this patient was excluded from the analysis. Another patient switched from ECX to EOF and then to EOX for unclear reasons and was also excluded from the analysis.

### Baseline Characteristics

Distribution of age and gender were similar. Eastern Cooperative Oncology Group (ECOG) performance status (PS) was significantly (*p* = 0.002) different in the two cohorts with superior PS in the ECX/EOX cohort. Forty-five percent of FLOT treated patients had PS 1 or worse compared to 28% in the ECX/EOX cohort. Patients in the FLOT cohort were significantly (*p* < 0.00001) more often staged by ^18^FDG PET-CT scans (95% vs. 32% in the ECX/EOX cohort). In the FLOT cohort, cN0 disease was statistically significant more common compared to ECX/EOX (70% vs. 36%, *p* < 0.00001). No differences in CT staging, the distribution of tumour location or frequency of signet ring cell carcinomas were recorded (Table 1).

**Table 1:** Baseline characteristics.

		ECX/EOX *n* = 186 (%)	FLOT *n* = 205 (%)	*p*
Median age (IQR)		65 (58–71)	66 (60–72)	0.18
Gender	Male	140 (74%)	163 (78%)	0.41
	Female	46 (26%)	44 (22%)	
Staging method	PET/CT scan	59 (32%)	193 (95%)	<0.00001
	CT scan	124 (67%)	9 (4%)	
	Other staging method	3 (2%)	1 (1%)	
Performance status	0	127 (68%)	109 (53%)	0.002
	1	47 (25%)	88 (43%)	
	2	6 (3%)	5 (2%)	
	Missing	6 (3%)	2 (1%)	
cT stage	T1	2 (1%)	2 (1%)	0.37
	T2	42 (23%)	65 (32%)	
	T3	111 (60%)	108 (53%)	
	T4	26 (14%)	25 (12%)	
	Tx	5 (3%)	4 (2%)	
cN stage	N0	67 (36%)	143 (70%)	0.00001
	N1-3	117 (63%)	59 (29%)	
	Nx	2 (1%)	2 (1%)	
Tumor location	Lower esophagus/GEJ	141 (76%)	172 (84%)	0.087
	Gastric	41 (22%)	32 (16%)	
	Missing	4 (2.1%)	0 (0%)	
Signet ring cell carcinoma	No	168 (90%)	189 (92%)	0.29
	Yes	12 (6.5%)	14 (6.8%)	
	Unknown	6 (3.2%)	2 (0.98%)	

### Efficacy outcomes

The primary endpoint of the study was not reached. pCR was recorded in 2.4 and 3.8% of all patients included in the FLOT and ECX/EOX cohorts, respectively (Table 2). There was no statistically significant difference in the distribution of ypT- or ypN-staging. The secondary endpoint for efficacy – margin-free regression – was met. R0 resection was achieved significantly more often both in all FLOT treated patients (86% vs. 78%, *p* = 0.03) and in those undergoing resection (95% vs. 88%, *p* = 0.05) compared to ECX/EOX. According to reports by the reading radiologist, numerically more patients treated with preoperative FLOT experienced a decrease in tumour size assessed by CT scans performed prior to surgery (56% vs. 48%, *p* = 0.087, Supplemental Table S1).

**Table 2:** Pathological assessment of resected patients including type of surgical procedure.

		ECX/EOX *n* = 186 (%)	FLOT *n* = 205 (%)	*p*
Resected (% of total)		163 (88%)	187 (91%)	0.25
Resection type	Total gastrectomy	26 (16%)	29 (16%)	0.55
	Subtotal gastrectomy	9 (5.5%)	8 (4.3%)	
	Esophagectomy w/o interposition	124 (76%)	144 (77%)	
	Esophagectomy+interposition	3 (1.8%)	4 (2.1%)	
	Esophagectomy+total gastrectomy+interposition	1 (0.61%)	2 (1.1%)	
	Unknown	0 (0%)	1 (%)	
R0 resection (% of total)		145 (78%)	177 (86%)	0.03
R0 resection (% of resected)		145 (89%)	177 (95%)	0.05
Complete pathological regression, ypT0N0 (% of resected)		7 (4.3%)	5 (2.7%)	0.69
Complete pathological regression, ypT0N0 (% of total)		7 (3.8%)	5 (2.4%)	0.58
ypT stage	≤T1	33 (20%)	36 (19%)	0.57
	T2	17 (10%)	28 (15%)	
	T3	101 (62%)	106 (57%)	
	T4	12 (7.3%)	17 (9%)	
ypN stage	N0	74 (45%)	76 (41%)	0.55
	N1	34 (21%)	39 (21%)	
	N2	23 (14%)	37 (20%)	
	N3	32 (20%)	35 (19%)	

**Figure 2:**
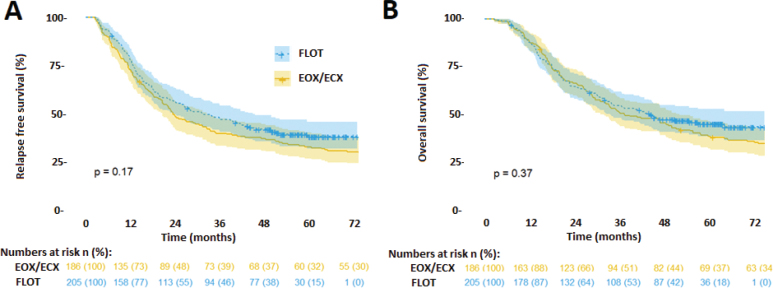
(A) Relapse-free survival and (B) Overall survival in the ECX/EOX (yellow) and FLOT (blue) cohort. 95% CI band is indicated as shaded area along each survival curve. Numbers of patients at risk with % in parentheses are indicated at bottom.

**Figure 3:**
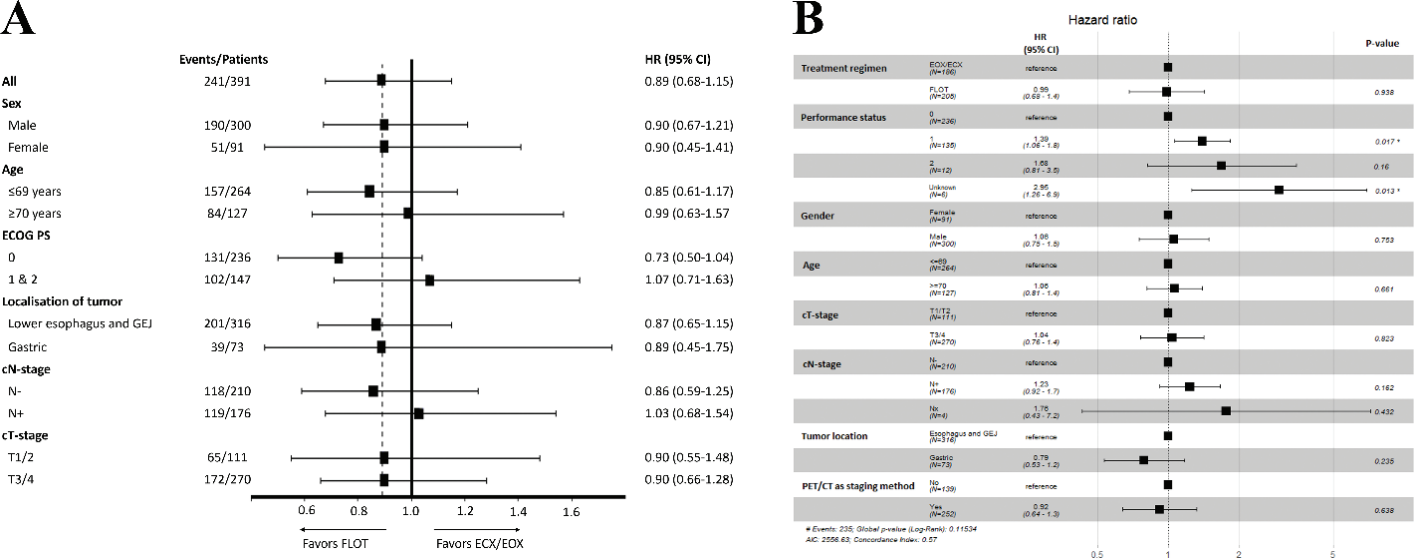
(A) Subgroup analysis. Forest plot of Cox proportional hazards analysis comparing overall survival between the ECX/EOX and FLOT cohort (B) Multivariate Cox Proportional Hazards regression model analysis of overall survival based on baseline characteristics of patients in the ECX/EOX and FLOT Cohort. Age Groups were categorised as patients ≤69 years and ≥70 years.

There was no significant difference (*p* = 0.17) in RFS between the ECX/EOX and FLOT cohorts, with a HR of 0.84 (95% CI: 0.66–1.08) in favour of FLOT. Similarly, there was no significant difference (*p* = 0.37) in OS, with a HR of 0.89 (95% CI: 0.68–1.15) in favour of FLOT (Figure 2). However, the FLOT cohort had significantly (*p* = 0.0006) shorter follow-up time with a mean of 37.7 compared to the ECX/EOX cohort with a mean follow-up of 47 months, preventing accurate assessment of long-term survival. The estimated OS at 2-, 3-, and 4-year were 65% (95% CI: 59–72), 54% (95% CI: 47–61), and 47% (95% CI: 41–55) months in the FLOT compared to 67% (95% CI: 60–74), 51 (95% CI: 44–59), and 44% (95% CI: 41–55) months in EXC/EOX group.Subgroup analysis (Figure 3) showed that patients with ECOG-PS 0 in the FLOT cohort trended toward better OS compared to patients with ECOG-PS 0 in the ECX/EOX cohort, HR = 0.73 (95% CI: 0.50–1.04). Multivariate Cox proportional hazards regression model for survival using baseline characteristics as variables did not indicate improved OS for the treatment regimen of FLOT, HR = 0.99 (95% CI: 0.68–1.4).

### Tolerability of chemotherapy

Discontinuations and dose reductions of drugs occurred equally often in the two treatment cohorts (Table 3). A larger number of patients in the FLOT cohort compared to ECX/EOX were admitted due to febrile neutropenia (11% vs. 3.8%, *p* = 0.0086) and patients in the FLOT cohort received G-CSF more frequently. More patients in the ECX/EOX cohort started on postoperative chemotherapy (65% vs. 48%, *p* = 0.0013), and more patients in the ECX/EOX cohort completed all pre- and postoperative chemotherapy cycles (39% vs. 28% p=0.022). Specific reasons for why patients discontinued perioperative chemotherapy and did not start postoperative chemotherapy can be seen in Supplemental Table S2 and Table S3, respectively.

**Table 3:** Tolerability and complications of chemotherapy and surgery.

		ECX/EOX *n* = 186 (%)	FLOT *n* = 205 (%)	*p*
			
Discontinuation of one or more drugs		39 (21%)	42 (21%)	0.91
Dose reduction		99 (53%)	110 (54%)	0.93
Febrile neutropenia		7 (3.8%)	22 (11%)	0.0086
G-CSF support		1 (0.53%)	142 (69%)	<0.00001
Started postoperative chemotherapy*		120 (65%)	99 (48%)	0.0013
Completion of chemotherapy#*		73 (39%)	58 (28%)	0.022
Clavien-Dindo*	0	110 (68%)	93 (50%)	0.008
	1	9 (6%)	18 (10%)	
	2	18 (11%)	27 (14%)	
	3	23 (14%)	41 (22%)	
	4	0 (0%)	6 (3%)	
	5	2 (1.2%)	2 (1.1%)	
Anastomotic leakage*		13 (8%)	12 (6%)	0.57
Median days admitted (IQR)		9 (8–13)	10 (9–15)	0.10
90-day mortality*		2 (1.2%)	0 (0%)	0.22

* % of resected patients;

# Completion is defined as administration of all scheduled cycles of chemotherapy regardless of dose reduction.

### Surgical complications

We found no difference in the type of surgery performed. There was a significant (*p* = 0.0082) difference in the distribution of surgical complications evaluated by Clavien-Dindo score with patients in the FLOT cohort experiencing more frequent and worse complications. There was no statistical difference in the rate of anastomotic leakage, days admitted to hospital after surgery or 90-day mortality after surgery (Table 3).

## Discussion

In a large, randomised trial, FLOT was proven the superior perioperative regimen in resectable esophagogastric ADC^9^. However, the clinical benefit of FLOT compared to ECX/EOX in an unselected real-world dataset is unknown. In this single-institution study, we planned to evaluate the effectiveness of FLOT in a retrospective cohort of consecutive patients. Endpoints were prespecified before the collection of data, with pCR selected as the primary endpoint based on large retrospective studies validating tumour regression grade as a robust surrogate for OS [13, 14]. Furthermore, survival in the FLOT arm of the FLOT4-AIO trial was associated with a significantly higher pCR rate [11]. The primary efficacy endpoint of the study was not met. pCR defined as ypT0N0 was observed in 2.7% of patients in the FLOT cohort compared to 4.3% in the ECX/EOX cohort. However, the observed pCR rates were considerably lower than expected according to the prespecified sample size estimation based on the FLOT4-AIO trial with an increase in pCR rate from 6 to 16% [11]. A contributing factor to the low pCR could be the histopathological examination of the entire GEJ used in our study compared to the assessment of the tumour bed only in the FLOT4-AIO trial. Furthermore, rates of pCR vary substantially in published reports. In one of the largest retrospective studies of tumour regression rate, the ypT0 rate reached only 3.4% (17/480) [13]. In contrast, pCR rates of 24 and 15% were reported in a recent randomised trial when pathology assessment was performed centrally compared to locally, indicating that the rate of pCR is dependent on the setting of the pathology assessment [15]. Still, the rate of pCR seems unexpectedly low in our study also when compared to a Danish nationwide retrospective study reporting a pCR rate of 6.3% [16].

The important secondary endpoint to assess efficacy – microscopic margin-free resection rate, R0, – also fulfils the criteria for a valid endpoint to assess benefit of treatment. For decades, achievement of R0 resection has been acknowledged as a bona fide and strong prognostic factor for survival based on large retrospective studies with more than 1,600 patients [17]. As for pCR, the FLOT4-AIO trial reported a significant increase in R0 resection rate in the FLOT arm indicating that R0 resection rate is a valid surrogate endpoint for patient outcome. In contrast to pCR, this validated secondary endpoint was reached. An R0 resection was achieved in 95% compared to 89% of resected patients (*p* = 0.05) and in 86% compared to 78% (*p* = 0.03) of patients who initiated preoperative FLOT and ECX/EOX, respectively. This is in line with the FLOT4-AIO trial reporting a significant increase in margin free resection in the intention-to-treat population from 78 to 85%.

HR for RFS and death were 0.84 and 0.89 in favour of FLOT, although with a *p*-value of only 0.17 and 0.37 respectively. In multivariate analysis of OS adjusting for baseline differences in prognostic factors HR for FLOT was only 0.99. Due to the shorter follow-up time in the FLOT cohort, assessment of benefit in long-time survival still awaits the maturation of survival data. Subgroup analysis showed that patients with a PS 0 score had trended towards better survival when treated with FLOT.

Accurate grading of toxicity induced by chemotherapy is challenging in retrospective studies as toxicity assessment using validated grading tools, for example, Common Terminology Criteria for Adverse Events is not done routinely outside clinical trials. However, tolerability of chemotherapy can be assessed by capturing readily recorded clinical decisions in response to toxicity such as rate of admission, dose delays, reduction and discontinuation of drugs.

As expected, compliance with chemotherapy was lower than that reported in the FLOT4-AIO trial as patients selected for clinical trials are more fit and staff conducting clinical trials are dedicated to ensure protocol adherence. In the entire population of the FLOT and ECX/EOX cohorts, a total of 80% completed preoperative chemotherapy, 56% started postoperative chemotherapy with only 29% completing postoperative chemotherapy compared to completion rates of 90, 56 and 41% at similar time points in the FLOT4-AIO trial. As opposed to the FLOT4-AIO trial, a significant lower proportion of patients completed FLOT than ECX/EOX. The reasons for discontinuation of chemotherapy varied between cohorts in a pattern comparable to the FLOT4-AIO trial; for example, diarrhoea and neuropathy resulted more often in cessation of FLOT than ECX/EOX including a significantly higher frequency of myelosuppression demonstrated by a significant difference in the rate of admission due to febrile neutropenia (10% vs. 3% in FLOT and EXC/EOX cohorts). This was despite the fact, that G-CSF support was almost exclusively used in FLOT treated patients. Nephrotoxicity more often led to cessation of ECX/EOX.

A significant increase in surgical complication rates was observed among FLOT treated patients. This observation should be interpreted with caution as the rate of 90-days mortality, anastomotic leakage and length of hospital stay were not different. In addition, in the FLOT4-AIO trial, no differences in the rate of surgical complications were reported.

Our study has several important limitations related to the retrospective design. An inherent weakness of retrospective studies are uncontrolled variations in disease management over time that can impact clinical outcome. A possible example of temporal factor that could affect our results, is the gradual implementation of ^18^FDG PET-CT scan as the preferred staging method. The vast majority of patients treated with FLOT were staged with ^18^FDG PET-CT scans in contrast to only a third of patients treated with ECX/EOX. The introduction of a more sensitive staging method is known to lead to the *Will Roger Phenomen*, that is, stage migration from lower to higher disease stages increasing stage specific survival rate without change in survival of the total population as reported in lung cancer^9^. Expectedly, preoperative ^18^FDG PET-CT scans could result in fewer resected patients as ^18^FDG PET-CT scans reduced futile thoracotomies in a randomised study in lung cancer mainly due to detection of metastatic disease [18]. A similar pattern was not evident in our data. Rather, a significant and marked increase in cN0 (36% vs. 78%, *n* = 0.0001) was recorded. The migration of cN stage did not result in change in yN stage. ^18^FDG PET-CT scans were not associated with downstaging of cT stage – a major determinant for achievement of R0 resection; survival was not significantly different in patients staged with ^18^FDG PET-CT scans compared to CT. Further, generalisability of our findings may be limited as our data are from a single institution.

A significant and considerably lower rate of PS 0 was recorded in the FLOT cohort compared to ECX/EOX treated patients. The reason for this difference is unclear. A shift in assessment of PS over time could explain the difference. However, if the observation reflects a true poorer PS among FLOT treated patients, it could potentially contribute to the recorded lower tolerability in the FLOT cohort. Furthermore, the higher PS in the ECX/EOX cohort, could result in a seemingly increased survival in this cohort and therefore potentially nullify or lessen differences in survival in favour of the FLOT treatment.

While large multicentre RCTs hold greater authority in establishing clinical evidence compared to non-randomized RWD, the collection and analysis of RWD are essential for validating RCT findings in routine clinical practice. RWD can reveal challenges when implementing practice changing therapies as demonstrated by our lower compliance and completion rates of FLOT compared to the FLOT4-AIO trial, highlighting potential limitations of intensive chemotherapy in unselected patient populations.

In conclusion, the primary endpoint of increased pCR rate in FLOT treated patients was not achieved. However, a higher rate of R0 resection was observed. Neither increased OS nor RFS could be demonstrated in the entire group of patients exposed to FLOT although subgroup analysis indicated a trend towards increased OS among patients with good performance status. A signal of poorer tolerability and slightly more frequent minor surgical complications were recorded for FLOT treated patients.

## Authors’ contributions

Authors TSA and KE collected data and undertook data analysis. MMS and LB conceived the project idea. MMS, LB and TSA prepared the outline of research project including the analysis plan. MMS, LB, MA, and MS supervised data collection. LB, MMS, RG, MA conducted review of pathological reports. All authors contributed to writing, editing, and final approval of the manuscript. The work reported in the paper has been performed by the authors, unless clearly specified in the text

## Data availability statement

The data that support the findings of this study are available from the corresponding author upon reasonable request from the authors

## Conflict of interest disclosure

The authors have no conflict of interests to declare.

## Ethical statement

The Danish Patient Safety Authority (Case no.: 31-1521-202) approved the collection of data from patient journals by the 26th of March 2020.

## Patient consent statement

As only retrospective data derived from medical charts were used, provision of informed consent was not required.

## Permission to reproduce material from other sources

Not relevant.

## Clinical trial registration

Not relevant.

## Supplementary Material

Implementation of perioperative FLOT compared to ECX/EOX chemotherapy regimens in resectable esophagogastric adenocarcinomas: an analysis of real-world data

Implementation of perioperative FLOT compared to ECX/EOX chemotherapy regimens in resectable esophagogastric adenocarcinomas: an analysis of real-world data
