# Supplementary material for: Implementation of perioperative FLOT compared to ECX/EOX chemotherapy regimens in resectable esophagogastric adenocarcinomas: an analysis of real-world data
Source: Acta Oncol. 2024 May 14;63:35431. doi: 10.2340/1651-226X.2024.35431 (PMC11332447; doi:10.2340/1651-226X.2024.35431)
Supplement: Implementation of perioperative FLOT compared to ECX/EOX chemotherapy regimens in resectable esophagogastric adenocarcinomas: an analysis of real-world data [file AO-63-35431-s1.pdf]

Supplementary material has been published as submitted. It has not been copyedited, or typeset by Acta Oncologica

Table S1 Change in tumor status by radiologic evaluation prior to surgery

The overall conclusion of the assessment of the reading radiologist is indicated. Scans have not been revised and the categorization of treatment effects do not comply with RECIST criteria P-value by chi-squared

	Increase	Decrease	Unchanged	Not evaluable	Unknown	Total	P-value
**FLOT**	9 (4.4%)	114 (56%)	59 (29%)	2 (0.98%)	21 (10%)	205	0.087
**ECX/EOX**	11 (5.9%)	90 (48%)	46 (25%)	2 (1.1%)	37 (20%)	186	

Table S2 Reasons for not completing chemotherapy

Table detailing reasons for not completing pre- and postoperative chemotherapy in the ECX/EOX and FLOT Cohort. More than one reason for failure to complete chemotherapy could be chosen. Values are listed as N unless otherwise stated)

Reason for not completing chemotherapy	Preoperative ECX/EOX n=29	Preoperative FLOT n=48	Postoperative ECX/EOX n=44	Postoperative FLOT n=39	Total ECX/EOX n=73	Total FLOT n=87
	n	n	n	n	n (% of 73)	n (% of 87)
**Cardiotoxicity**	2	6	2	1	4 (5,5%)	7 (8.1%)
**Febrile neutropenia**	1	2	1	1	2 (2.8%)	3 (3.5%)
**Hem tox other than febrile neutropenia**	7	7	7	1	14 (19%)	8 (9.1%)
**Neuropathy**	0	6	0	16	0	22 (25%)
**Diarrhea**	4	11	4	4	8 (11%)	15 (17%)
**Hand-and-foot syndrome**	2	0	2	4	4 (5.5%)	4 (4.6%)
**Decreasing performance status**	4	14	4	12	8 (11%)	26 (30%)
**Nausea and/or vomiting**	9	11	9	9	18 (25%)	20 (23%)
**Thromboembolic events**	1	1	1	0	2 (2.7%)	1 (1.2%)
**Hepatotoxicity**	1	1	1	0	2 (2.7%)	1 (1.2%)
**Progression**	1	1	1	0	2 (2.7%)	1 (1.2%
**Nephrotoxicity**	2	1	2	0	4 (5.5%)	1 (1.2%
**Patient request**	5	7	5	7	10 (14%)	14 (16%)

Table S3 Reasons for not starting postoperative chemotherapy

Table for reasons for not starting postoperative chemotherapy for patients in the ECX/EOX and FLOT cohort. More than one reason for not starting postoperative chemotherapy could be chosen. Values are listed as n (Percentage of total patients)

	**ECX/EOX**	**FLOT**
**Underwent Surgery but did not start postoperative chemotherapy**	n=43	n=99
**Patients wish**	13 (30%)	20 (20%)
**Inadequate Performance status**	7 (16%)	34 (34%)
**Toxicity during past chemotherapy**	7 (16%)	22 (22%)
**Physicians' decision**	3 (7%)	12 (12%)
**Age**	1 (2.3%)	0 (0%)
**Frailty**	8 (19%)	10 (10%)
**Comorbidity**	1 (2.3%)	0 (0%)
**Tumor resistant to chemotherapy**	2 (4.7%)	4 (4%)
**Other**	4 (9.3%)	9 (9%)
**Unknown**	0 (0%)	1 (1%)
