# Supplementary figures and images for: Implementation of perioperative FLOT compared to ECX/EOX chemotherapy regimens in resectable esophagogastric adenocarcinomas: an analysis of real-world data
Source: Acta Oncol. 2024 May 14;63:35431. doi: 10.2340/1651-226X.2024.35431 (PMC11332447; doi:10.2340/1651-226X.2024.35431)

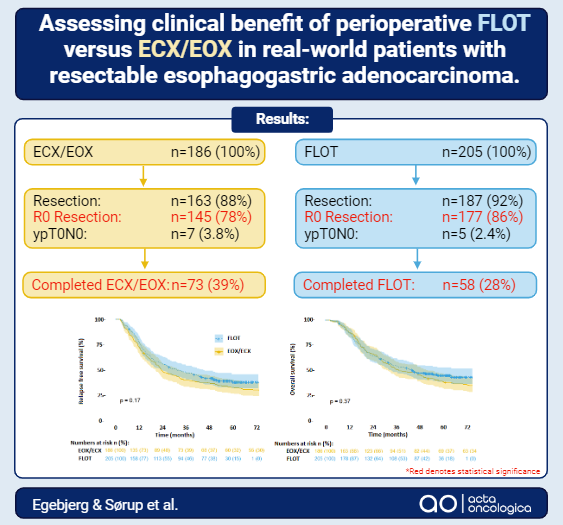

Supplement: Implementation of perioperative FLOT compared to ECX/EOX chemotherapy regimens in resectable esophagogastric adenocarcinomas: an analysis of real-world data [file AO-63-35431-s2.png]
